# Identifying markers of biofilm formation on medical-grade stainless steel as a representative medical device material

**DOI:** 10.1099/mic.0.001684

**Published:** 2026-04-01

**Authors:** Gregory G. Anderson, Sravya Kovvali, Francis W. Dang, Ryan M. Singh, Apoorva Vishwakarma, Jon W. Weeks, Ruchi Pandey

**Affiliations:** 1 Division of Biology, Chemistry, and Materials Science, Center for Devices and Radiological Health (CDRH), Office of Science and Engineering Laboratories, US Food and Drug Administration (FDA), Silver Spring, MD 20993, USA; 2 Division of Health IB, CDRH, Office of Product Evaluation and Quality, Office of Health Technology I, FDA, Silver Spring, MD 20993, USA

**Keywords:** biofilm, medical device, reprocessing

## Abstract

Reusable medical devices are reprocessed between uses, including cleaning and, as necessary, disinfection or sterilization. Healthcare-associated infections have been attributed to reusable medical devices and linked to inadequate reprocessing, which can result in residual soil on the device, insufficient disinfection, microbial resistance to used disinfectants and biofilm-related contamination. These factors can lead to microbial proliferation on and biofouling of reusable medical devices, increasing the risk of patient infection. While there are FDA-recognized standards for cleaning validation (including artificial test soils), there is a lack of standards or guidance documents available to advise on determining whether biofilm has been adequately cleaned off reusable devices after reprocessing. Additionally, relatively few studies report reproducible models of biofilm formation on medical devices or device materials; such models are necessary to begin the identification of the microbial biofilm burden present before and after reprocessing. Moreover, appropriate analytes to quantify that biofilm burden, and the endpoints of those analytes after reprocessing, need to be determined. The study described herein utilized a drip flow reactor (DFR) to develop single-species biofilms of two Gram-negative (*Pseudomonas aeruginosa* and *Klebsiella pneumoniae*) and two Gram-positive (*Staphylococcus aureus* and *Enterococcus faecalis*) bacterial species that are prone to contaminate medical devices as biofilms. Biofilm was extracted at early and late biofilm stages and then tested for several analytes, including protein, ATP, endotoxin, peptidoglycan and total organic carbon. The levels of these analytes were compared to c.f.u. and metabolic activity to qualitatively compare analyte levels with biofilm burden. The results presented demonstrate that the DFR can be used to model biofilm formation of several medically relevant micro-organisms on stainless steel. Furthermore, the analytical data obtained with this study indicate that the analytes used can be a good starting point for informing the selection of endpoints in future studies that evaluate the efficacy of cleaning and disinfection within the context of biofilm reduction.

## Introduction

Reusable medical devices require reprocessing between patient uses to ensure safety and reduce the risk of infection. During clinical use, these devices may become contaminated with patient-derived materials, environmental residues, procedure-related substances (e.g. lubricants) and micro-organisms. Reprocessing therefore includes cleaning as a critical initial step, followed by disinfection or sterilization as appropriate for the device and its intended use [1,2]. Regulatory guidance documents and consensus standards provide frameworks for manufacturers to develop and validate reprocessing instructions included in device labelling [3,8], and healthcare facilities are expected to implement these instructions as part of routine practice. To verify the effectiveness of cleaning processes, established standards define acceptance criteria for residual soils such as protein, total organic carbon (TOC), carbohydrate, haemoglobin and ATP [7].

Despite these reprocessing procedures, infections stemming from contaminated medical devices are common [9,11]. Such healthcare-associated infections (HAIs) have been associated with gastroscopes, bronchoscopes, duodenoscopes, colonoscopes, respiratory equipment, surgical instruments and other reusable devices [11,17]. The annual medical cost for treatment of device-related HAIs is $8–11 billion in the USA alone [18,19], and these infections account for tens of thousands of deaths each year [11,12]; a large part of that healthcare burden is attributed to contaminated reusable devices [11]. Moreover, HAIs frequently involve antimicrobial-resistant micro-organisms, which are prevalent in the healthcare environment [11,12]. The micro-organisms contaminating medical devices may originate from the environment, the patient and/or the healthcare worker. Persistence of microbes on the device surfaces can result from incomplete or inadequate reprocessing, contamination after reprocessing (such as from rinse water) or the formation of surface-associated microbial communities known as biofilms, among other factors [11,12].

Biofilm formation in particular is a major cause of device-related infections [12,15,20]. Clinical biofilms on medical devices frequently consist of multispecies communities, in which interspecies interactions can influence biofilm architecture, metabolic activity and tolerance to cleaning and disinfection processes [21]. Biofilms form when micro-organisms initially attach reversibly to a surface (such as a medical device), followed by aggregation and differentiation into a structured community that becomes increasingly irreversibly attached as it matures and is surrounded by an extracellular matrix of nucleic acids, proteins and carbohydrates [22]. On medical devices, the source of the biofilm micro-organism could be the patient, the healthcare worker or the environment. The nature of the biofilm confers upon the constituent bacteria an extreme tolerance to antimicrobials, detergents and biocides, such as cleaners and disinfectants used during reprocessing [12,14, 20]. Furthermore, a build-up of biofilm layers on medical devices has been seen after multiple rounds of reprocessing in some cases, wherein the bacteria are protected under a shell of chemically fixed extracellular matrix and patient material [23,26]. This build-up of biofilm can be extremely difficult to eliminate.

Though biofilm formation on medical devices poses a great threat of both infection and transmission of this infection, there is a lack of guidance documents or standards to advise on determining whether biofilm has been adequately removed from reusable devices. Moreover, there are relatively few studies reporting reproducible models of biofilm formation on medical devices or device materials. Such a model is necessary to begin to identify the microbial biofilm burden present before and after reprocessing, as well as to determine the appropriate analytes to quantify that biofilm burden and the endpoints of those analytes after reprocessing. Previous work has demonstrated the successful use of standardized reactor systems such as the Centers for Disease Control and Prevention Biofilm Reactor (CDC-BR) and drip flow reactor (DFR) for developing reproducible biofilms on stainless steel medical device materials [27]. This prior work established optimized parameters for biofilm growth and extraction, serving as a foundation for further work to identify biofilm markers. Establishing reproducible laboratory biofilm models and baseline biofilm-associated markers is a necessary prerequisite to subsequent studies evaluating the effectiveness of cleaning and disinfection strategies.

To address these concerns, this study utilized a DFR [28,29] to develop single-species biofilms of four bacteria (two Gram-negative and two Gram-positive) that are prone to contaminate medical devices as biofilms: *Pseudomonas aeruginosa*, *Klebsiella pneumoniae*, *Staphylococcus aureus* and *Enterococcus faecalis* [17,30,32]. At early (6 h) and late (48 h) biofilm stages, biofilm was extracted and then tested for levels of several analytes, including protein, ATP, endotoxin, peptidoglycan and TOC. The levels of these analytes were compared to c.f.u. and metabolic activity to establish baseline relationships between analyte levels and biofilm burden, which may inform future investigations of biofilm removal during cleaning and disinfection.

## Methods

### Bacterial strains and growth conditions

This study used *P. aeruginosa* ATCC 15442, *K. pneumoniae* ATCC 33495, *S. aureus* ATCC 12600 and *E. faecalis* ATCC 33186. All strains were grown in tryptic soy broth (TSB) overnight at 37°C, with shaking, or streaked out on tryptic soy agar (TSA) plates and grown overnight at 37°C.

### Biofilm formation using DFR

Biofilms for the four test organisms were developed on 18.75 cm^2^ stainless steel coupons (7.5×2.5 cm, #316, brush finish #4, 1.2 mm thick; McMaster Carr) using six-chambered polyethylene terephthalate DFRs (BioSurface Technologies, Bozeman, MT) as per ASTM E2647-20 [29], with minor adaptations. Coupons were cut to size in-house from a stainless steel sheet. Coupons were placed in each chamber of a DFR base, and the entire DFR base was then wrapped in aluminium foil and autoclaved. Once cooled, the autoclaved DFR was placed in a biological safety cabinet and aseptically unwrapped. Subsequently, autoclaved ½″ silicone tubing was attached to the DFR effluent ports and then clamped to prevent leakage of the inoculum. Inoculum was prepared by growing overnight cultures of test bacteria in TSB. Overnight cultures were diluted into fresh medium (3 g l^−1^ TSB for Gram-negative organisms or 30 g l^−1^ TSB for Gram-positive organisms) to an OD at 600 nm (OD_600_) of 0.2–0.4, corresponding to ~8.0×10⁷ to 9.0×10⁸ c.f.u. ml^−1^. For each DFR channel, 15 ml of the appropriate sterile medium and 1 ml of the diluted culture were aseptically added to achieve a final inoculum volume of 16 ml and a target cell density of 5×10⁶ to 6×10⁷ c.f.u. ml^−1^. One channel on each DFR utilized received 16 ml sterile TSB (3 or 30 g l^−1^, depending on the organism tested in the other chambers), to serve as the negative control. After chamber inoculation, the channel lids were replaced and 0.2 µm pore filters were attached as previously described [29]. The reactor system was then incubated at 35°C (for Gram-positive organisms) or room temperature (18–27°C; for Gram-negative organisms) for 6 h; this static incubation is the batch phase (Table S1, available in the online Supplementary Material).

After completion of the batch phase, the DFR base (with attached effluent tubes) was tilted and held at a 10° angle using the DFR stand. A total of 0.27 g l^−1^sterile TSB (for Gram-negative organisms) or 3 g l^−1^ sterile TSB with 2 g l^−1^ glucose (for Gram-positive organisms), prepared in 10 l carboys, was placed nearby to be used as the source of fresh medium for the experiment. This medium was supplied to each DFR chamber via a MasterFlex IPC peristaltic pump (VWR). Size 16 silicone tubing was attached to each medium carboy; then, Y connectors were used to split the medium flow into six lines (of size 16 silicone tubes). Each of these medium lines was then connected (via straight connectors) to 2-stop Tygon S3 2.79 mm (inner diameter) pump tubes (Masterflex, VWR), which were each connected to individual pump channels. The other end of the pump tubing was connected (via straight connectors) to size 16 silicone tubing. Twenty-one-gauge needles attached to the end of these media lines were inserted into the DFR Mininert™ valves, to deliver medium to the top of each coupon. Similarly, size 18 silicone tubing (Masterflex, VWR) was used to connect the effluent tubing to a waste carboy to discard the spent media from each channel. The effluent tubes were unclamped, and the pump was activated to deliver the sterile fresh media to each channel of the DFR at a flow rate between 0.92 and 0.75 ml min^−1^; this continuous flow phase was sustained for 42 h at room temperature (18–27°C) (Table S1). This flow rate was selected in accordance with ASTM E2647-20 to maintain low-shear conditions that support reproducible biofilm development rather than to simulate a specific clinical flow environment.

### Biofilm extraction

After the continuous phase, sterile disposable forceps (Fisher Scientific) were used to retrieve the coupons. Planktonic cells were rinsed off the coupon by gentle dipping into a 50 ml conical tube filled with sterile deionized water. The rinsed coupons were subsequently scraped into a total of 50 ml sterile deionized water using a sterile reusable Policeman (Bel-Art™ SP Scienceware™ PTFE Policeman, Fisher Scientific) to extract the biofilm sample; the 50 ml water was added in 10–15 ml aliquots during the scraping procedure to assist in rinsing material off the coupon into the collection vessel. Sterile deionized water was used as the extraction medium to avoid interference with downstream analytical assays; prior work demonstrated no significant difference in c.f.u. recovery when biofilms were extracted in water compared to 1× PBS under these conditions [27]. A Fisherbrand™ 150 Handheld Homogenizer Motor (Fisher Scientific) with sterile probe (Fisher Scientific) was used to homogenize the extracted sample for 30 s at 20,500±5,000 r min^−1^.

### Analytical assays

#### C.f.u.

Biofilm extracts were serially diluted in sterile water and plated on TSA plates. The use of water for serial dilution was selected to maintain consistency with the extraction medium and to avoid interference with downstream assays; prior work demonstrated no significant impact on c.f.u. recovery when water was used instead of buffer under these conditions [27]. After overnight growth at 37°C, c.f.u. were counted. C.f.u. were then multiplied by the extraction volume and divided by the coupon surface area to derive c.f.u. cm^−2^ of the coupon.

#### Metabolic activity

Metabolic activity of the biofilm was quantified using alamarBlue™ cell viability reagent (Molecular Probes) [33,35]. One hundred microlitres of the samples was added in duplicate to a 96-well black-walled microtitre plate (Nunc, Fisher); fresh extraction medium was used as a blank. Ten microlitres of alamarBlue™ reagent was then added to each well, and the plate was incubated, protected from light, for 2 h at 37°C. After incubation, fluorescence was measured using a Tecan M1000 spectrophotometer set to excitation and emission wavelengths of 570 and 585 nm, respectively.

#### Confocal microscopy analysis

For every DFR experiment, two coupons were prepared for confocal microscopy analysis. After rinsing, as above, the microscopy coupons were treated with 200 µl of live/dead stain (FilmTracer LIVE/DEAD Biofilm Viability Kit, Invitrogen) prepared according to the manufacturer’s instructions, and the coupons were then incubated in the dark at room temperature for 30 min. The treated coupons were then rinsed again via gentle dipping into a 50 ml conical tube filled with sterile water. The coupon was then held vertically, and excess water was wicked away from the edge using laboratory task wipes. Afterwards, three drops of mountant (ProLong Glass Antifade Mountant, Invitrogen) were dispensed onto the coupon’s biofilm-cultivating surface. Immediately thereafter, a coverslip was carefully placed on top of the coupon to avoid trapping air bubbles beneath the glass. Coupons were then allowed to cure in the dark at room temperature for 60 h, and then, they were stored in Petri dishes wrapped in foil at 4°C until imaging. Prepared coupons were imaged using a Leica TCS SP8 X confocal microscope. To capture both stains, the excitation and emission profiles for EGFP and propidium iodide (PI) were chosen for the Syto 9 and PI in the live/dead stain, respectively. The microscope was then adjusted to place the biofilm in focus, and a z-stack was taken to capture the entire biofilm depth. Ten different locations on each coupon were imaged.

#### Protein quantification by BCA assay

Protein was quantified using the Microplate bicinchoninic acid (BCA) Protein Assay Kit (Thermo Scientific – Pierce). Standards, ranging between 250 and 3.9 µg ml^−1^, were prepared by diluting the BSA stock (2 mg ml^−1^), provided by the kit, into sterile deionized water (the extraction medium). Nine microlitres of each standard and sample was tested, in duplicate, in a 96-well flat-bottom microplate (Falcon) according to the manufacturer’s instructions. The absorbance was measured at 562 nm using a Tecan M1000 spectrophotometer. The sample protein concentration was determined from the standard curve.

#### Protein quantification by o-phthaldialdehyde assay

The o-phthaldialdehyde (OPA) protein quantification assay was performed using the Fluoraldehyde™ OPA Reagent Solution (Thermo Scientific) or Fluoraldehyde OPA Crystals (Fisher Scientific), according to the manufacturers’ instructions. BSA standards, between 250 and 3.9 µg ml^−1^, were prepared as above. Next, 20 µl of standard, blank (sterile deionized water) or analyte/samples were added in duplicate to a 96-well black-walled plate (Nunc, Fisher), after which 200 µl of the Fluoraldehyde Reagent Solution was added to each well using a multichannel pipette. The fluorescence was measured within 5 min of adding the reagent solution using a Tecan M1000 spectrophotometer set to an excitation wavelength of 340 nm and an emission wavelength of 455 nm. The sample protein concentration was determined from the standard curve.

#### ATP quantification

ATP levels within the biofilm were measured via ATP Determination Kit (Fisher Scientific Invitrogen). Briefly, 10 ml of reaction solution was prepared by adding 8.9 ml deionized H_2_O, 0.5 ml 20× reaction buffer, 0.1 ml 0.1 M DTT, 0.5 ml 10 mM d-luciferin and 2.5 µl 5 mg ml^−1^ firefly luciferase. This solution was then gently inverted to mix, minimizing exposure to light by covering with aluminium foil during preparation and handling. One hundred microlitres of the prepared reagent was added to individual wells of a white-walled 96-well plate (Nunc, Fisher); the total number of wells each receiving this 100 µl was equivalent to the number of standards and samples tested in duplicate. The background luminescence was then measured in a Tecan M1000 spectrophotometer. Ten microlitres of ATP standards and samples was added to each well, and the plate was incubated on the bench for 2–5 min, protected from light. The luminescence was then measured in the same spectrophotometer. ATP concentrations were then calculated by subtracting background luminescence from each well, plotting a standard curve and using linear regression to determine the ATP concentrations of each sample.

#### Total organic carbon

The TOC content of the biofilm extracts was measured using a Hach QP1680 High-Temperature TOC Laboratory Analyzer (with auto sampler, 96 positions). The analyser was configured to measure non-purgeable organic carbon (NPOC). A total volume of 10 ml was used per sample. The samples used for the analyses were heat-treated by incubation at 100°C for 10 min and were either used undiluted (Gram-positive organisms) or diluted 1:10 v/v with sterile deionized water (for Gram-negative organisms). Organic carbon levels were analysed according to the manufacturer’s instructions for NPOC.

#### Endotoxin quantification

Total endotoxin levels within biofilm were determined by the use of a PyroSmart NextGen Recombinant limulus amebocyte lysate (LAL) kit (Associates of Cape Cod, Inc.). Briefly, biofilm extracts were diluted to between 1:50 and 1:100 in endotoxin-free water (to obtain measurable range). Twenty microlitres of these samples, along with 20 µl of undiluted negative control and 20 µl of standards, was added to a 384-well plate (VWR). Three minutes before use, the lyophilized LAL reagent was resuspended in 2.8 ml of room-temperature reconstitution buffer, swirled gently for 1 min and incubated at room temperature for 2 min at room temperature. Promptly, 20 µl of reconstituted reagent was added to each well; then, the plate was transferred into a Tecan M1000 spectrophotometer preheated to 37°C. Absorbance was measured at 405 nm every 30 s for 1 h. Endotoxin levels were determined by generating a logarithmic standard curve and using this curve to calculate endotoxin units (EU) in the samples.

#### Peptidoglycan quantification

Peptidoglycan content of the biofilm was determined by a SLP-HS Single Reagent Set II kit (Fujifilm Wako Chemicals). Briefly, 20 µl of peptidoglycan standards and samples was added to a 384-well plate (VWR). To these samples, 20 µl of resuspended SLP reagent (resuspended according to the manufacturer’s instructions) was added, after which absorbance at 430 nm was measured every 40 s for 1.5 h at 30°C in a Tecan M1000 spectrophotometer. Peptidoglycan concentrations were then determined via generation of a logarithmic plot of the standard data and subsequent generation of a second order polynomial curve from this plot.

### Statistical analysis

Three independent biofilm experiments (biological replicates) were performed for each micro-organism, with three replicate samples per experiment (technical replicates). C.f.u. values were log_10_-transformed prior to statistical analysis. Statistical comparisons were performed within each bacterial species, comparing early (6 h) and mature (48 h) biofilms using two-tailed Student’s t-tests. No interspecies or multiple-group comparisons were performed, and therefore, no multiple-comparison corrections were applied. A *P* value <0.05 was considered statistically significant. Formal statistical correlation analyses between analyte levels and c.f.u. were not performed; relationships described reflect comparative trends observed across biofilm growth stages and species.

## Results

### Microbial enumeration of biofilm extracts

**Fig. 1.**
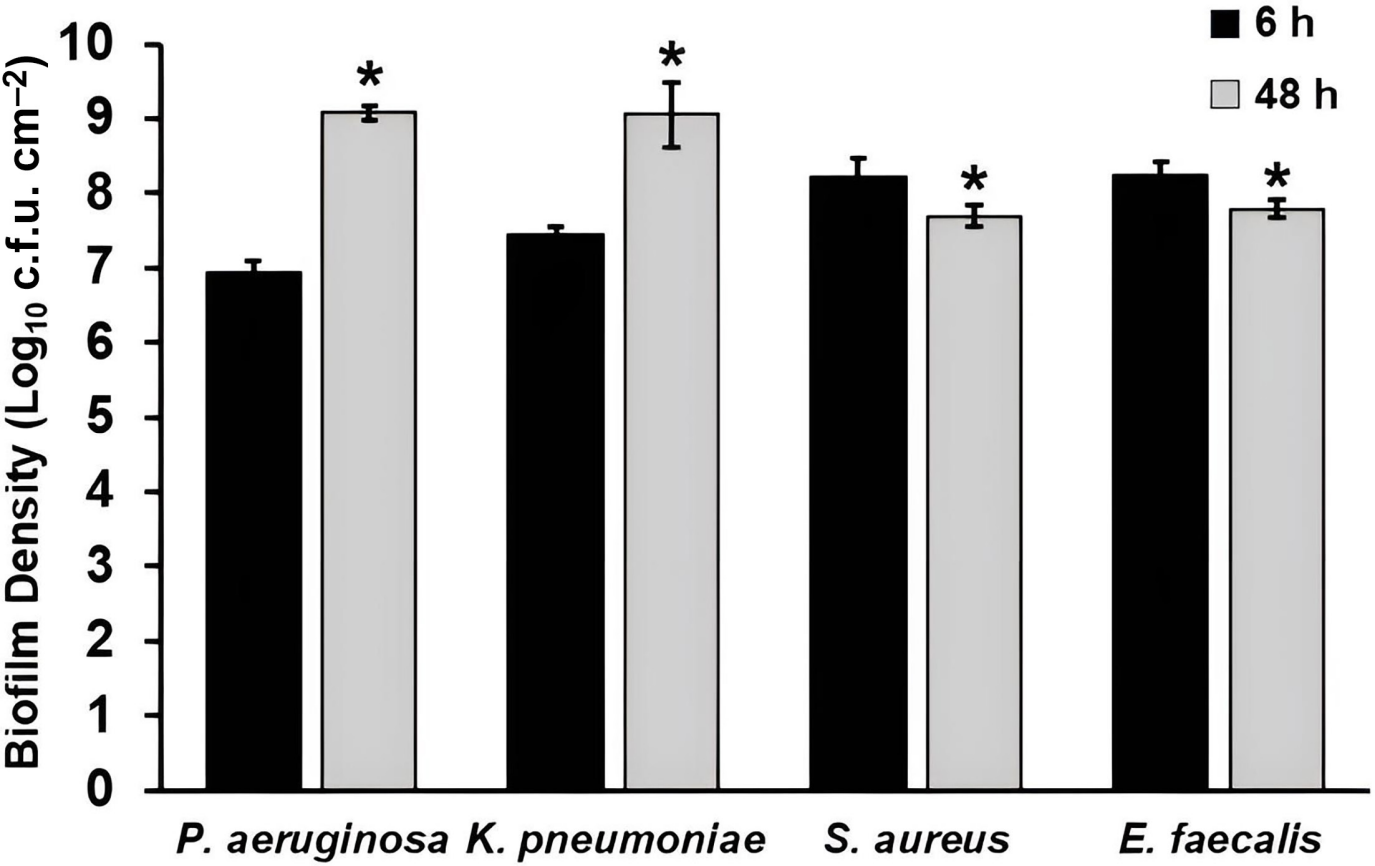
Biofilm density of *P. aeruginosa*, *K. pneumoniae*, *S. aureus* and *E. faecalis* after batch phase only (6 h) and after batch and continuous phase (48 h). *P<0.05, compared to 6 h. Error bars represent mean±sd from three independent biological replicates. Data for multiple species are shown together for visual comparison; statistical analyses were performed within species only.

**Fig. 2.**
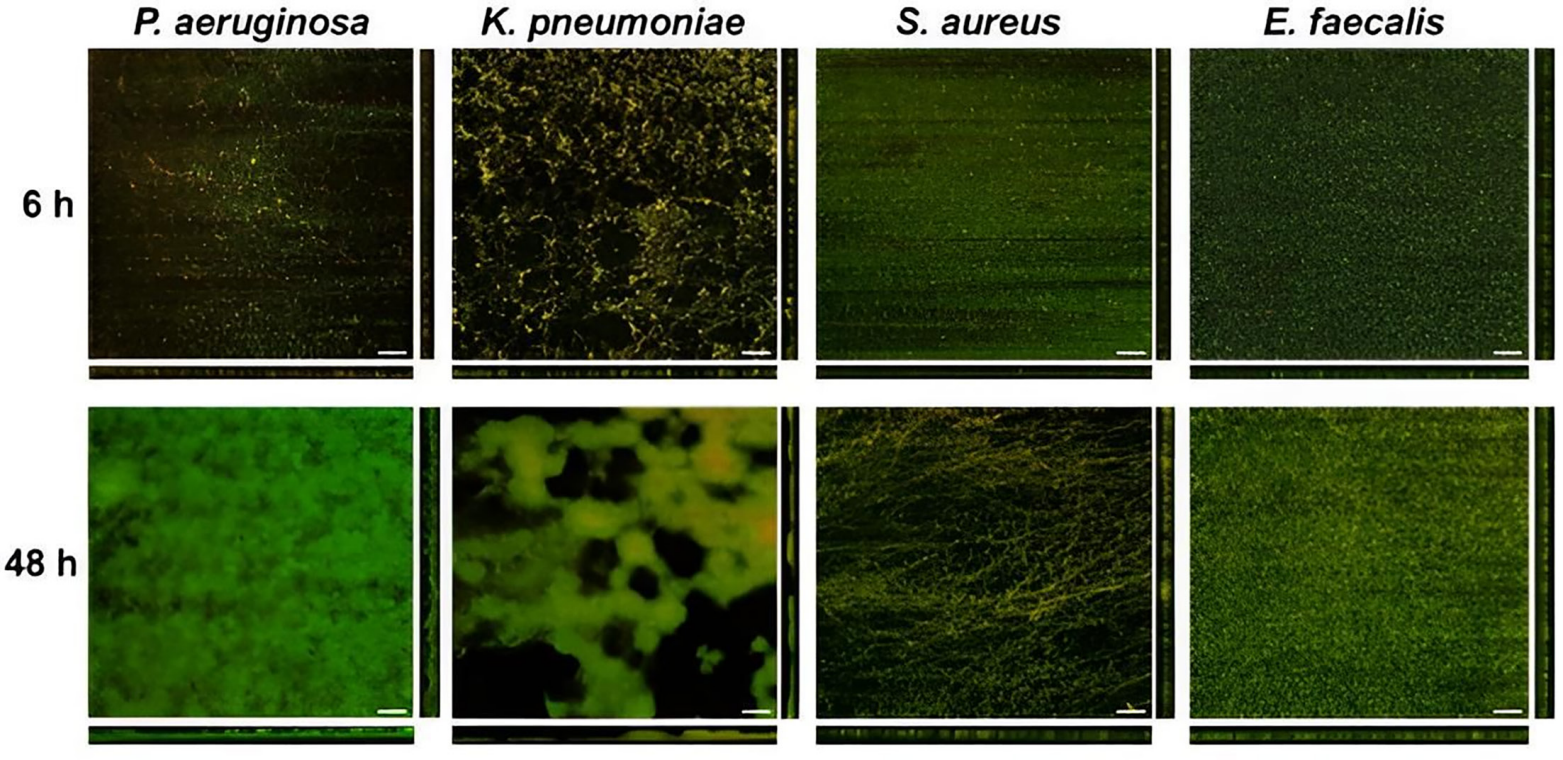
Confocal micrographs of *P. aeruginosa*, *K. pneumoniae*, *S. aureus* and *E. faecalis* after batch phase only (6 h) and after batch and continuous phase (48 h). Biofilms were stained with FilmTracer LIVE/DEAD Biofilm Viability kit, as described in Methods. Scale bars=50 µm.

The DFR system was used to develop single-species biofilms of *P. aeruginosa*, *K. pneumoniae*, *S. aureus* and *E. faecalis* for a total of 48 h (6 h of batch phase plus 42 h of continuous flow). All species were inoculated into the DFR at similar cell densities (Table S1). Using separate sets of coupons for each timepoint, we extracted biofilm after the 6 h batch phase and after the full 48 h of biofilm growth, to quantify biofilm levels at early and late timepoints, respectively. Accordingly, all results presented compare early (6 h) and mature (48 h) biofilms formed under identical conditions and do not include evaluation of cleaning efficacy or comparisons to cleaned or non-inoculated coupons. Extracted biofilm was serially diluted and plated for c.f.u. enumeration. Clear differences were observed between the Gram-negative and Gram-positive bacteria tested. At 6 h, *P. aeruginosa* and *K. pneumoniae* biofilm densities were ~7 log_10_ c.f.u. cm^−^² and increased to ~9 log_10_ c.f.u. cm^−^² by 48 h (Fig. 1). For both species, the increase in c.f.u. was significantly higher at 48 h, compared to 6 h (*P*<0.05). Confocal microscopy images reflect these c.f.u. counts (Fig. 2); by 48 h, biofilm was thicker and exhibited much greater surface coverage than at 6 h. Additionally, *P. aeruginosa* and *K. pneumoniae* developed biofilms distinct from each other. More microcolonies were apparent on *K. pneumoniae* coupons at 6 h, versus *P. aeruginosa* coupons, reflecting the ~3-fold greater c.f.u. of *K. pneumoniae* (9.01×10^6^ vs 2.79×10^7^ c.f.u. cm^−2^, respectively; see Fig. S1). At 48 h, *P. aeruginosa* biofilms almost completely covered the coupon, while *K. pneumoniae* biofilms were patchy (Fig. 2).

The two Gram-positive bacteria tested, *S. aureus* and *E. faecalis*, showed a very different pattern. Biofilm levels at 6 h reached ~8 log_10_ c.f.u. cm^−2^ but dropped slightly to 7.7–7.8 log_10_ c.f.u. cm^−2^ by 48 h (Fig. 1). Confocal microscopy showed a redistribution of micro-organisms between 6 and 48 h, wherein cells appear homogenously spread across the surface at 6 h but more clustered by 48 h (Fig. 2).

**Fig. 3.**
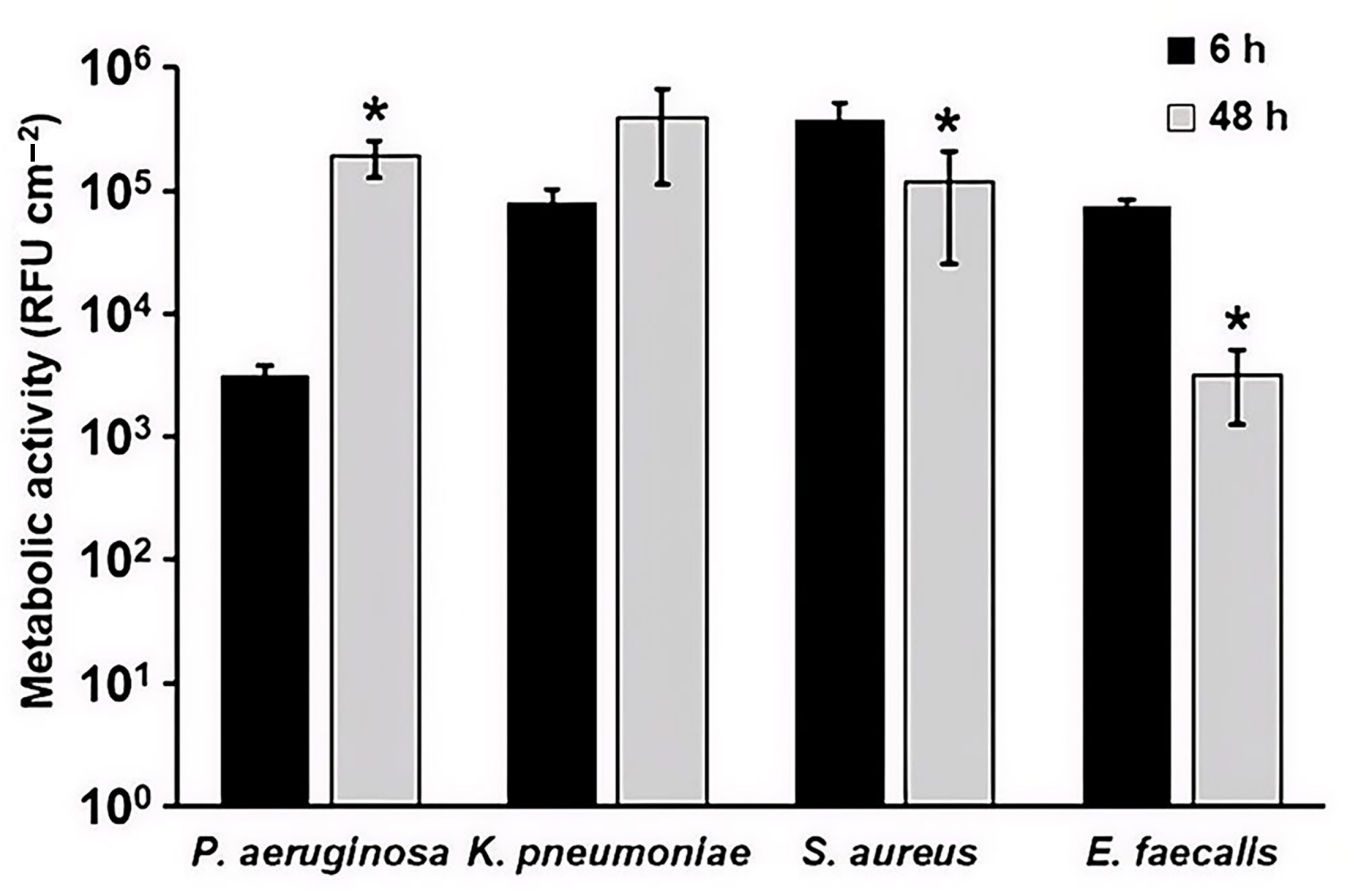
Metabolic activity in *P. aeruginosa*, *K. pneumoniae*, *S. aureus* and *E. faecalis* biofilm extracts after batch phase only (6 h) and after batch and continuous phase (48 h). *P<0.05, compared to 6 h. Error bars represent mean±sd from three independent biological replicates. Data for multiple species are shown together for visual comparison; statistical analyses were performed within species only.

In addition to c.f.u. and confocal microscopy, biofilms were assessed using alamarBlue™ to determine the metabolic activity of the bacteria within the biofilms [33,35]. *P. aeruginosa* biofilm metabolic activity increased significantly (~2 log increase in relative fluorescence units) from 6 to 48 h (Fig. 3). *K. pneumoniae* also exhibited an increase (~5-fold) over time, but the difference was not significant. *S. aureus* and *E. faecalis* metabolic activity significantly decreased from 6 to 48 h, and *E. faecalis* activity was less than *S. aureus* at both timepoints (Fig. 3).

#### Quantification of protein in biofilm extracts

**Fig. 4.**
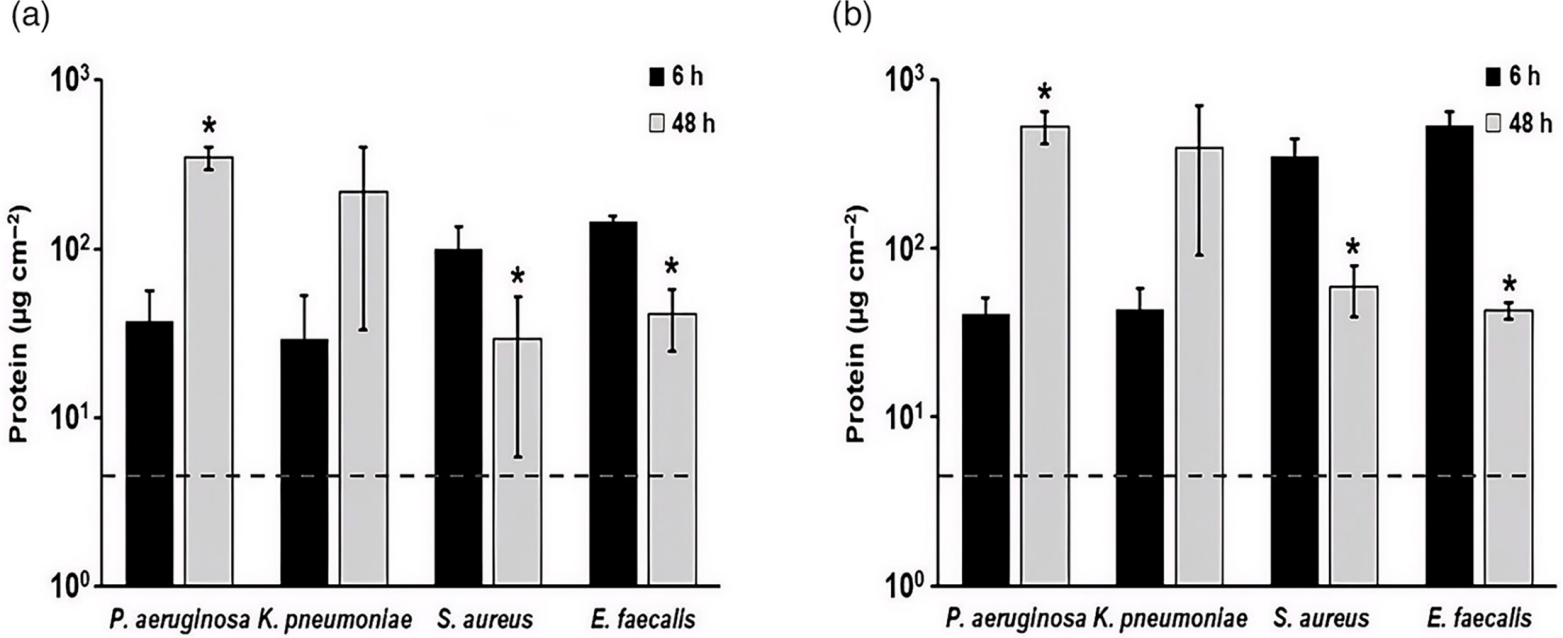
Total protein concentration in *P. aeruginosa*, *K. pneumoniae*, *S. aureus* and *E. faecalis* biofilm extracts after batch phase only (6 h) and after batch and continuous phase (48 h) quantified using microplate (**a**) BCA assay and (**b**) using OPA assay. The two assays differ in detection chemistry and sensitivity, which contributes to differences in measured protein levels. *P<0.05, compared to 6 h. Error bars represent mean±sd from three independent biological replicates. Data for multiple species are shown together for visual comparison; statistical analyses were performed within species only. The dashed horizontal line indicates the AAMI/ANSI ST98 acceptance limit for residual protein (6.4 µg cm^−2^).

Protein is a common marker quantified to assess reprocessing efficiency [7]. Consequently, protein was measured in DFR biofilm samples by both BCA assay and OPA assay. Both *P. aeruginosa* and *K. pneumoniae* exhibited nearly tenfold increases in protein level between 6 and 48 h, as quantified by both BCA (Fig. 4a) and OPA (Fig. 4b) assays. On the other hand, *S. aureus* and *E. faecalis* both exhibited a decrease in protein levels. In the BCA assay, both micro-organisms’ protein levels decreased by approximately one third. In the OPA assay, *S. aureus* protein levels decreased ~86% and *E. faecalis* protein levels decreased ~94%.

#### Quantification of ATP in biofilm extracts

**Fig. 5.**
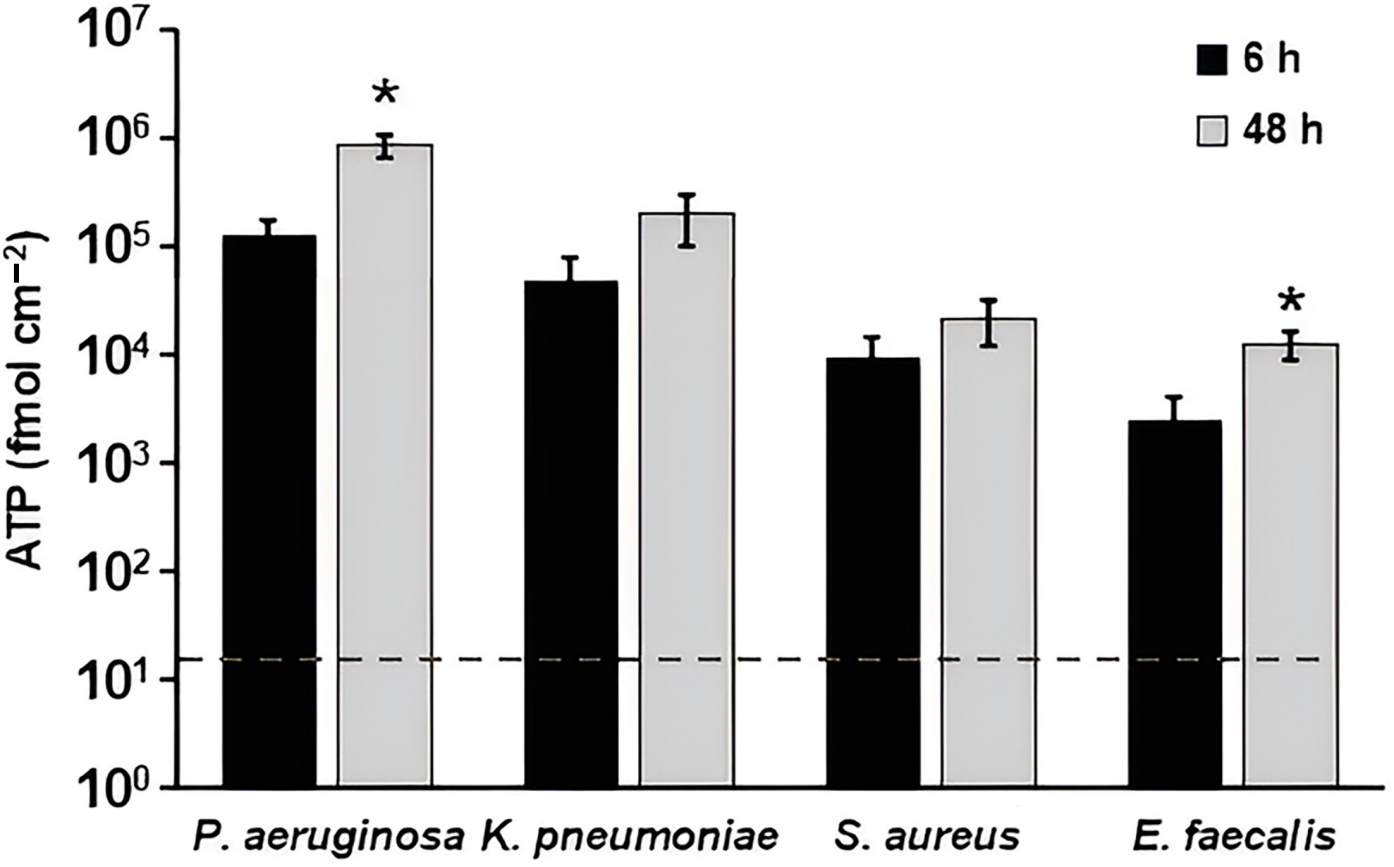
ATP concentration in *P. aeruginosa*, *K. pneumoniae*, *S. aureus* and *E. faecalis* biofilm extracts after batch phase only (6 h) and after batch and continuous phase (48 h). *P<0.05, compared to 6 h. Error bars represent mean±sd from three independent biological replicates. Data for multiple species are shown together for visual comparison; statistical analyses were performed within species only. The dashed horizontal line indicates the AAMI/ANSI ST98 acceptance limit for residual ATP (22 fmol cm^−2^).

Another analyte commonly assayed for reprocessing efficiency is ATP [7]. A statistically significant increase in the ATP concentration was observed in the biofilm extracts of *P. aeruginosa* and *E. faecalis* from the 6 h to the 48 h timepoint (*P*<0.05) (Fig. 5). ATP levels also increased from 6 to 48 h in *K. pneumoniae* and *S. aureus* biofilm extracts, but these differences were not significant (*P*>0.05). Additionally, the ATP concentrations of the two Gram-negative bacteria were approximately tenfold higher than the ATP levels from the two Gram-positive bacteria tested.

#### Quantification of TOC

**Fig. 6.**
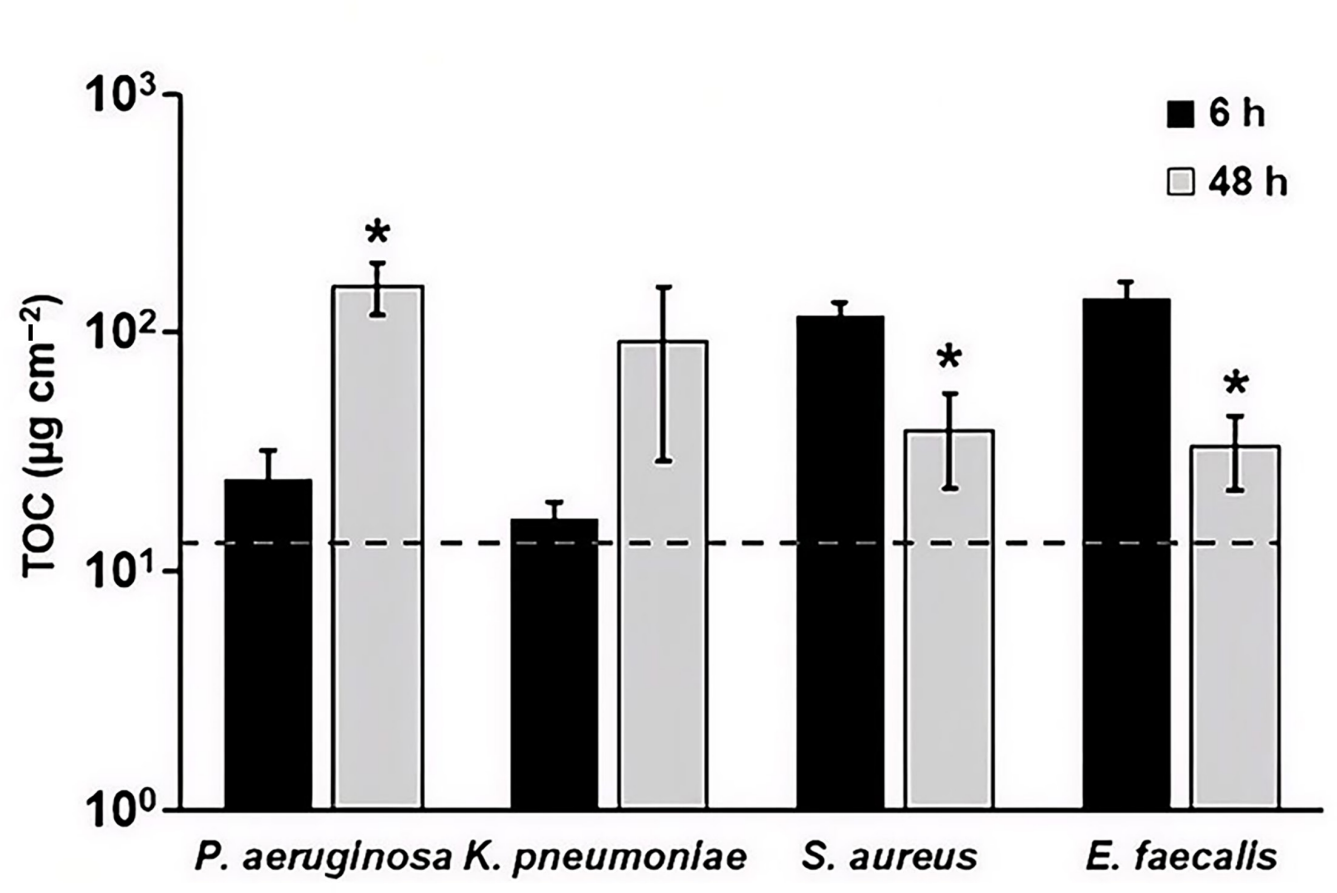
TOC concentration in *P. aeruginosa*, *K. pneumoniae*, *S. aureus* and *E. faecalis* biofilm extracts after batch phase only (6 h) and after batch and continuous phase (48 h). *P<0.05, compared to 6 h. Error bars represent mean±sd from three independent biological replicates. Data for multiple species are shown together for visual comparison; statistical analyses were performed within species only. The dashed horizontal line indicates the AAMI/ANSI ST98 acceptance limit for TOC (12 µg cm^−2^).

TOC is also commonly quantified as a cleaning marker for reprocessing validation [7]. For the Gram-negative bacteria (*P. aeruginosa* and *K. pneumoniae*), an increase in the levels of TOC was observed in biofilm extracts from the 6 h to the 48 h timepoint (Fig. 6). This increase was statistically significant for *P. aeruginosa* biofilm extracts (*P*<0.05). On the other hand, for Gram-positive bacteria (*S. aureus* and *E. faecalis*), a significant decrease in the levels of TOC was observed in biofilm extracts from the 6 h to the 48 h timepoint (*P*<0.05) (Fig. 6).

#### Quantification of microbial-specific markers – endotoxin and peptidoglycan – in biofilm extracts

While protein, ATP and TOC are commonly used markers in medical device cleaning validation, they are not specific to micro-organisms. In the present study, these analytes reflect biofilm-associated material because no additional soil was introduced. However, under clinical use conditions, such analytes may also originate from patient material, procedure-related additives or environmental sources, limiting their specificity as biofilm indicators.

**Fig. 7.**
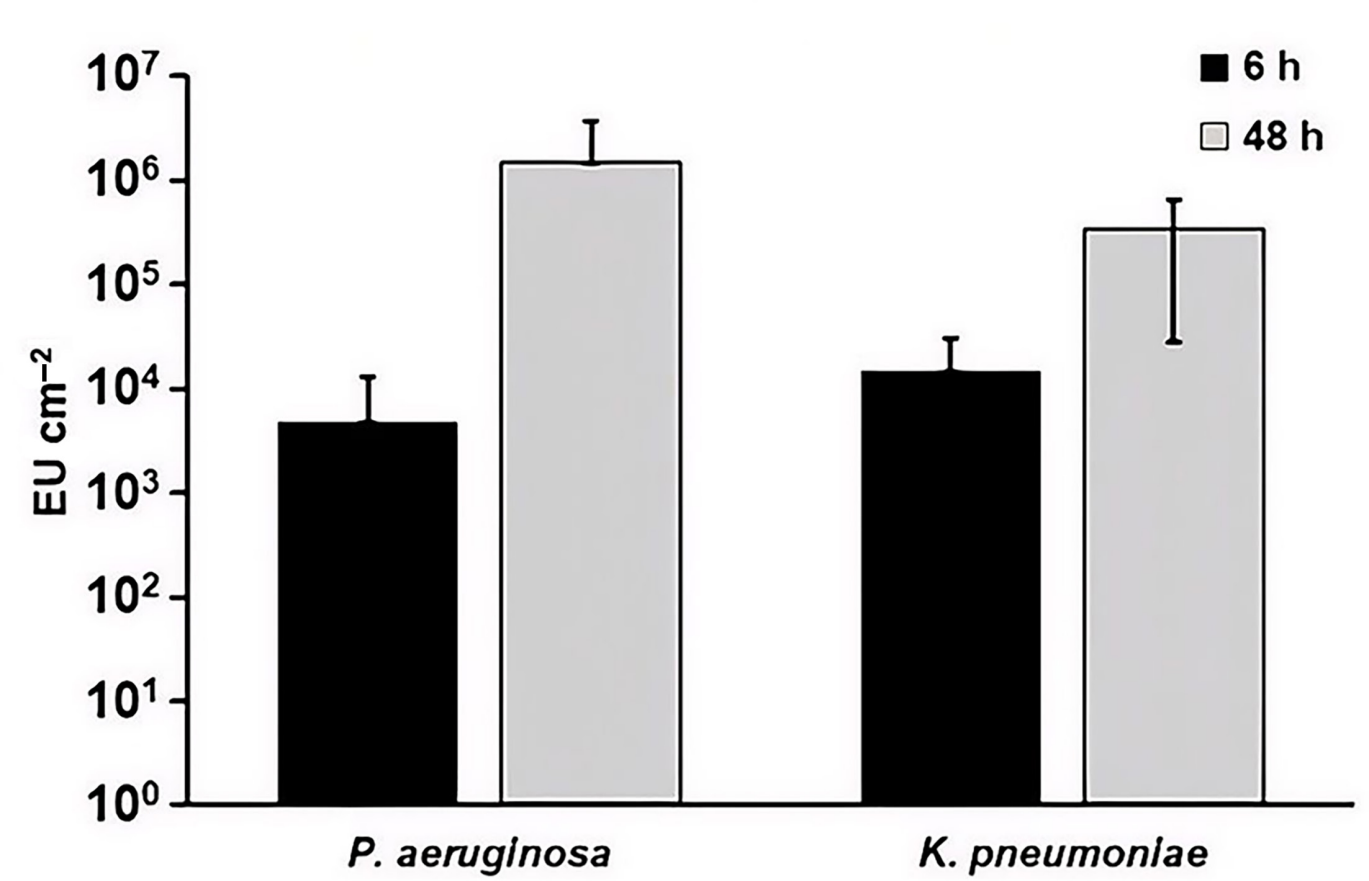
EU in *P. aeruginosa* and *K. pneumoniae* biofilm extracts after batch phase only (6 h) and after batch and continuous phase (48 h). Differences between 6 and 48 h were not statistically significant within either species (*P*>0.05). Error bars represent mean±sd from three independent biological replicates. Data for multiple species are shown together for visual comparison; statistical analyses were performed within species only. The assay limits of detection for endotoxin (see Table S2) fall below the plotted range and are therefore not shown. No AAMI/ANSI ST98 acceptance limit exists for endotoxin.

To complement these non-specific markers, bacterial-specific analytes were also evaluated. Endotoxin, a component of the LPS layer of Gram-negative bacteria, was detected only in *P. aeruginosa* and *K. pneumoniae* biofilms and increased from 6 to 48 h, although this increase was not statistically significant (Fig. 7). As expected, endotoxin levels were negligible in *S. aureus* and *E. faecalis* biofilms.

**Fig. 8.**
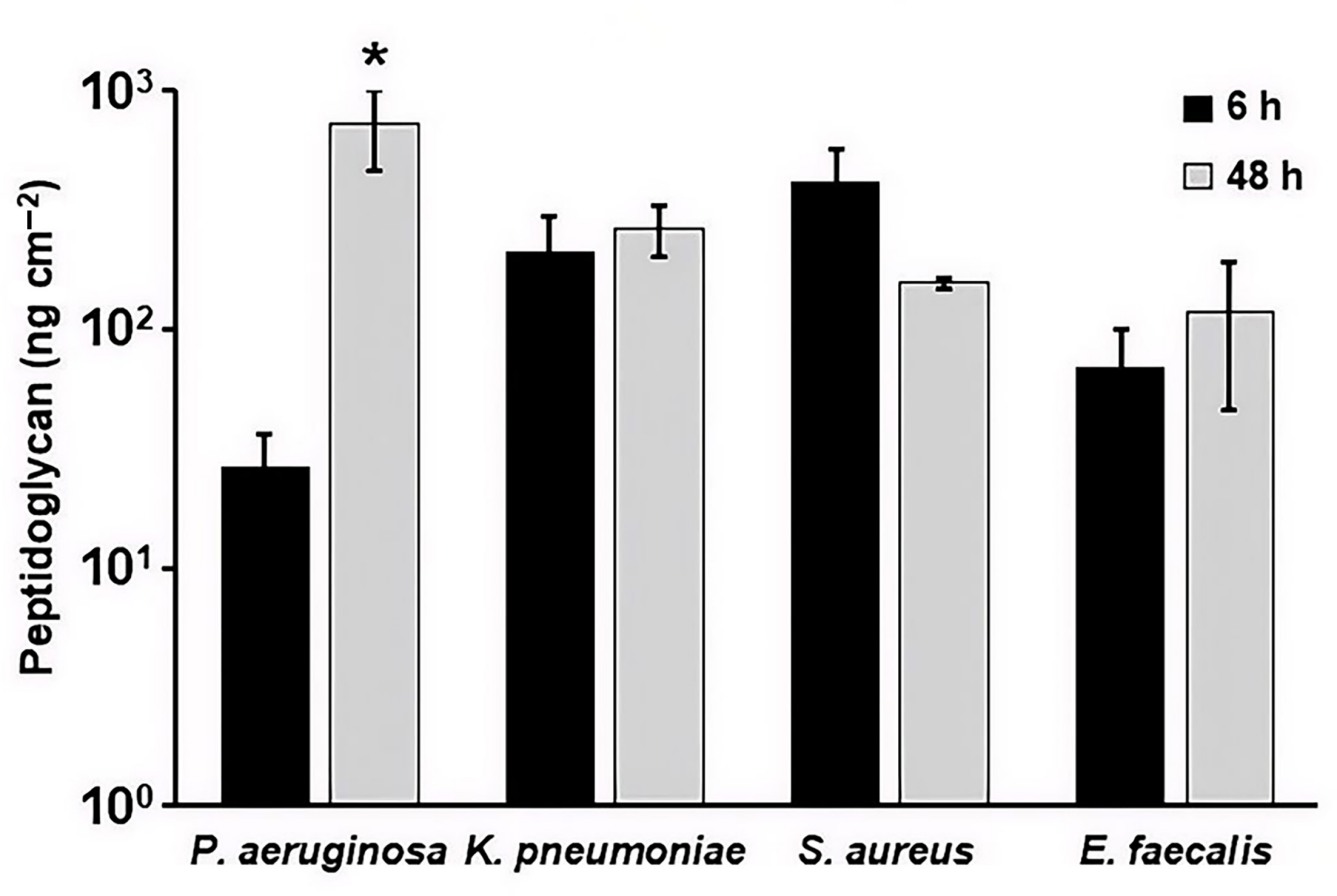
Peptidoglycan concentration in *P. aeruginosa*, *K. pneumoniae*, *S. aureus* and *E. faecalis* biofilm extracts after batch phase only (6 h) and after batch and continuous phase (48 h). *P<0.05, compared to 6 h. Error bars represent mean±sd from three independent biological replicates. Data for multiple species are shown together for visual comparison; statistical analyses were performed within species only. The assay limit of detection for peptidoglycan (see Table S2) falls below the plotted range and is therefore not shown. No AAMI/ANSI ST98 acceptance limit exists for peptidoglycan.

Peptidoglycan, which is present in both Gram-negative and Gram-positive bacteria, was also assessed. Peptidoglycan levels increased significantly in *P. aeruginosa* biofilms at 48 h relative to 6 h (Fig. 8), while smaller, non-significant changes were observed for the other species.

#### Summary

A summary of all the DFR experiments is found in Table S1 and Fig. S1. All experiments were inoculated with similar c.f.u. and were carried out under equivalent conditions. Though each species established biofilms of a different c.f.u. level, the marker levels were generally commensurate with the c.f.u. of that species (Fig. S1). That is, relative c.f.u. amounts between the species are reflected in the relative marker amounts between the species. For all analytical assays, the lowest value obtained for each was above the limit of detection (LOD) for that respective assay, apart from BCA (Table S2). Additionally, all values were above the AAMI/ANSI ST98 indicated limits for cleaning [7] (Table S2).

## Discussion

The results reported herein indicate that biofilms of medically relevant bacteria readily form on stainless steel in the DFR. This work was intended to characterize biofilm formation and associated analytical signals under controlled conditions, rather than to directly assess the effectiveness of cleaning or reprocessing procedures. While the Gram-negative organisms tested (*P. aeruginosa* and *K. pneumoniae*) showed similar growth patterns to each other and the Gram-positive organisms tested (*S. aureus* and *E. faecalis*) showed similar growth patterns to each other, biofilm development over time significantly differed between the Gram-negatives and the Gram-positives (Figs 1, 2 and 3). That is, while *P. aeruginosa* and *K. pneumoniae* increased in c.f.u. and metabolic activity from 6 to 48 h, *S. aureus* and *E. faecalis* began with high c.f.u. and metabolic activity at 6 h, followed by a modest decline by 48 h. Species-dependent biofilm growth kinetics and time-dependent fluctuations in viable counts have been reported previously and can reflect differences in nutrient availability, dispersal and maturation-associated physiological changes, which may vary across organisms and growth conditions. Consistent with this, prior studies of *S. aureus* and *E. faecalis* biofilms have reported temporal fluctuations rather than a monotonic increase under certain experimental conditions [36,39]. Similar condition-dependent biofilm dynamics have also been described for Gram-negative species, including *P. aeruginosa*, in which biomass and viability can change with maturation and dispersal depending on hydrodynamic and nutrient parameters. Accordingly, the Gram-negative versus Gram-positive patterns observed here may be interpreted in the context of the standardized DFR growth conditions used in this study.

Another main goal of this research, in addition to developing a model for forming biofilms of medically relevant bacteria on medical device materials, was to identify markers of those biofilms that could be used in future studies to assess reprocessing efficacy. AAMI/ANSI ST98 indicates markers that are recommended to be quantified to assess the removal of patient soil during cleaning, as well as endpoints for those markers that shall not be exceeded: protein (≤6.4 µg cm^−2^), TOC (≤12 µg cm^−2^), carbohydrate (≤1.8 µg cm^−2^), haemoglobin (≤2.2 µg cm^−2^) and ATP (≤22 fmol cm^−2^) (see Table S2). Likewise, to be able to make reprocessing claims with respect to biofilm, it will be important to determine markers and endpoints for cleaning and disinfection of biofilm ‘soil’.

Using AAMI/ANSI ST98 as a guide and starting point for identifying useful biofilm markers, protein, TOC and ATP levels were analysed in this study (Figs 4, 5 and 6). Carbohydrate and haemoglobin were excluded; only a small number of bacteria produce haemoglobins [40,41], and the level of carbohydrate in biofilm extracellular matrix is highly variable between species [42,47]. As shown in Fig. S1, we found that DFR biofilms resulted in protein, ATP and TOC levels that were above the ST98 limits (see Table S2). This finding is important in that it demonstrates that biofilm formation, even at low levels (Fig. S1, Table S2), results in analyte levels outside the acceptance criteria for devices considered clean and ready for reuse. Thus, it should be possible to distinguish between analyte levels before and after cleaning and disinfection of biofilm-soiled device materials. Additionally, most of these marker levels were much greater than the LOD of the respective assays. However, protein levels of lower-density biofilms were approaching or below the LOD of the assays.

Differences in the magnitude of error bars observed across bacterial species reflect a combination of biological variability and species-specific biofilm characteristics rather than methodological inconsistency. Biofilm formation is inherently heterogeneous, and variability in extracellular matrix composition, biofilm architecture and detachment behaviour among species can contribute to differences in analyte recovery between biological replicates. In addition, species-dependent differences in growth dynamics and biofilm maturation may amplify variability at later time points. Importantly, all assays were performed using standardized protocols across experiments, and variability was consistent across independent biological replicates, indicating that the observed differences in error bar magnitude primarily reflect real biological differences rather than limitations in assay reproducibility.

The significance of these results on identifying marker endpoints during biofilm cleaning and disinfection is unclear. It is expected that cleaning and disinfection will result in c.f.u. that is orders of magnitude lower than the lowest c.f.u. reported herein for the organisms tested. With this caveat in mind, it is possible that protein might be a poor analyte for testing residual biofilm post-reprocessing. A more thorough investigation is needed on this topic, including the potential use of a more sensitive protein assay. Conversely, these data suggest that ATP and TOC may serve as candidate biofilm-associated markers for assessing biofilm presence under the controlled conditions used in this study.

Markers that specifically identify the presence of bacteria were also tested: endotoxin and peptidoglycan (Figs 7 and 8). These molecules are found in the outer layers of bacteria. The values obtained from biofilms are much greater than the LOD, so these analytes might also be useful for detecting any residual biofilm after cleaning and disinfection. However, there is little evidence as to what endpoints would be clinically meaningful or allow for disinfection/sterilization steps of reprocessing to remain effective. In addition, the utility of these markers varies by organism and assay. Endotoxin is specific to Gram-negative bacteria; therefore, an endotoxin test cannot detect Gram-positive biofilms [13,22, 48]. Thus, the absence of endotoxin does not necessarily imply the absence of biofilm, particularly for Gram-positive organisms. Peptidoglycan detection may also be species-dependent and influenced by differences in cell wall structure, biofilm composition and assay specificity [21,22]. Moreover, measurement of peptidoglycan may be less practical for routine cleaning assessment due to the need for specialized reagents and potential background contributions and is therefore best considered a complementary or research-focused marker rather than a standalone indicator [49].

Interpretation of biofilm-associated markers following cleaning and disinfection further depends on how different reprocessing steps affect individual analytes. ATP and protein levels can be reduced or eliminated by detergents, disinfectants or high-temperature washing processes, which may lead to diminished marker signals even in the presence of residual biofilm material [50,52]. In contrast, endotoxin is relatively heat stable and may, as previously mentioned, persist after bacterial death, such that its detection may reflect prior Gram-negative biofilm contamination rather than viable organisms [48,51]. Similarly, peptidoglycan reflects bacterial cell wall material and may persist following microbial inactivation, limiting its ability to distinguish between viable and non-viable biofilm residues [21,22]. Taken together, these observations indicate that changes in marker signals following cleaning reflect distinct biological and chemical attributes of biofilms and are most informative when interpreted in the context of the cleaning mechanism employed and in combination with other markers.

Analyte quantification showed a trend similar to the biofilm growth patterns (Fig. S1). For Gram-negative bacteria, levels of analytes (protein, ATP, TOC, peptidoglycan and endotoxin) increased from 6 to 48 h (though not significantly for *K. pneumoniae*), while analytes in Gram-positive biofilms generally decreased over this time period. Notably, ATP levels in the Gram-positive biofilm extracts increased from 6 to 48 h despite modest declines in c.f.u. over the same period. Similar divergences between viability-based measures and ATP-based signals have been reported in biofilm systems and may reflect time-dependent physiological shifts during maturation, including changes in cellular energy state and the proportion of metabolically active subpopulations within the community [13,14]. In addition, ATP-based readouts can be influenced by factors such as biofilm architecture and extraction efficiency, as well as the relative contributions of intracellular versus extracellular ATP in mature biofilm matrices [49]. Accordingly, the Gram-negative versus Gram-positive patterns observed here are best interpreted in the context of the standardized DFR growth conditions used in this study. Microscopy further indicates a maturation process for all four species tested (Fig. 2). For *S. aureus* and *E. faecalis*, structural changes are apparent in the biofilm, even though c.f.u. decreases.

Additionally, while *K. pneumoniae* biofilm growth (c.f.u.) demonstrated a significant increase from 6 to 48 h, increases in analytical assay values were not statistically significant. We speculate that variability in biofilm formation observed across technical and biological replicates contributed to the lack of statistically significant differences in some analytical assays. C.f.u. data were log_10_-transformed for statistical analysis, as is standard practice for microbial count data, to normalize distributions and reduce the influence of extreme values; raw c.f.u. values exhibited similar qualitative trends but greater dispersion (data not shown).

Another notable observation with the analytical assays is that there was an apparent 1.4- to 4-fold increase in OPA-based protein concentrations over BCA-based protein concentrations when determined for the same samples, apart from *E. faecalis* samples at 48 h, which experienced a decrease (Fig. 4). The reason for this difference in protein levels between the methods is likely related to differences in detection chemistry of the assays, as has been described previously [53]. However, except for *S. aureus* and *E. faecalis* at 6 h, none of these differences between protein values were significant.

There were several limitations of this study. First, only stainless steel coupons were used. Biofilm formation on additional medically relevant materials will thus need to be tested in future work. Additionally, the data presented are specific to the four bacterial species used for biofilm formation. Though the bacteria investigated are common contaminants of medical devices and represent a range of the bacterial domain, there are many other species that can contaminate medical devices. This study is also limited to the analytes tested; other analytes could yield different patterns between timepoints. Another limitation of this study is that non-inoculated coupons and post-cleaning samples were not included, as the focus was on defining baseline biofilm-associated signals across organisms and growth stages. Inclusion of negative controls and cleaned coupons will be an important component of future studies aimed at directly evaluating biofilm removal during cleaning and disinfection.

Finally, a limited number of data points, representing a limited number of biofilm densities, were tested. An increase in the number of different biofilm densities tested would be needed to increase the predictive correlation between c.f.u. and analytes.

As mentioned, protein, ATP and TOC (along with carbohydrates and haemoglobin) have been frequently used to assess medical device cleaning validation [7]. Additionally, c.f.u., protein, ATP, TOC and endotoxin have been used to assess cleaning efficacy in clinical settings [50,52]. Studies have also used c.f.u., protein, ATP, TOC and/or endotoxin to quantify bacterial biofilm on medical device-related or other surfaces [48,49, 54,56]. However, this is the first report, to our knowledge, to quantify all these analytes simultaneously within the context of biofilms on medically relevant material surfaces. Overall, the results presented demonstrate that the DFR can be used to model biofilm formation of several medically relevant micro-organisms on stainless steel.

Under the controlled conditions used in this study, changes in ATP, TOC, peptidoglycan, endotoxin and protein levels tracked qualitatively biofilm development across growth stages and species, suggesting that these analytes represent candidate biofilm-associated markers that may inform future studies evaluating residual biofilm following cleaning and disinfection. Protein measurements at lower biofilm densities approached assay detection limits, indicating that assay sensitivity may influence the detection of low-level residual biofilm. The findings of this study expand upon previous work, establishing reproducible DFR and CDC-BR biofilm models on stainless steel [27]. Whereas the previous study focused on reactor comparison and optimization, the present work identifies key biofilm analytes that could serve as practical markers for evaluating biofilm reduction following cleaning and disinfection. Differences between bacterial species should be considered.

## Supplementary material

10.1099/mic.0.001684 Uncited Supplementary Material 1.

## Abbreviations

CDC-BR: Centers for Disease Control and Prevention Biofilm Reactor
DFR: drip flow reactor
EU: endotoxin units
HAI: healthcare-associated infection
LAL: limulus amebocyte lysate
LOD: limit of detection
NPOC: non-purgeable organic carbon
OPA: o-phthaldialdehyde
PI: propidium iodide
TOC: total organic carbon
TSA: tryptic soy agar
TSB: tryptic soy broth

## References

[1] Spaulding E, Lawrence CBS, Lawrence CBS (1968). Disinfection, Sterilization, and Preservation.

[2] Kremer T, Rowan NJ, McDonnell G (2024). A proposed cleaning classification system for reusable medical devices to complement the Spaulding classification. J Hosp Infect.

[7] (2022).

[9] Houri H, Aghdaei HA, Firuzabadi S, Khorsand B, Soltanpoor F (2022). High prevalence rate of microbial contamination in patient-ready gastrointestinal endoscopes in Tehran, Iran: an alarming sign for the occurrence of severe outbreaks. Microbiol Spectr.

[10] FDA Use duodenoscopes with innovative designs to enhance safety: FDA safety communication. https://www.fda.gov/medical-devices/safety-communications/use-duodenoscopes-innovative-designs-enhance-safety-fda-safety-communication2022.

[11] Mishra A, Aggarwal A, Khan F (2024). Medical device-associated infections caused by biofilm-forming microbial pathogens and controlling strategies. Antibiotics (Basel).

[12] Bouhrour N, Nibbering PH, Bendali F (2024). Medical device-associated biofilm infections and multidrug-resistant pathogens. Pathogens.

[13] Caldara M, Belgiovine C, Secchi E, Rusconi R (2022). Environmental, microbiological, and immunological features of bacterial biofilms associated with implanted medical devices. Clin Microbiol Rev.

[14] Donlan RM (2001). Biofilms and device-associated infections. *Emerg Infect Dis*.

[15] Jamal M, Ahmad W, Andleeb S, Jalil F, Imran M (2018). Bacterial biofilm and associated infections. J Chin Med Assoc.

[16] Karvouniaris M, Brotis A, Tsiakos K, Palli E, Koulenti D (2022). Current perspectives on the diagnosis and management of healthcare-associated ventriculitis and meningitis. Infect Drug Resist.

[17] Mehta AC, Muscarella LF (2020). Bronchoscope-related “Superbug” infections. Chest.

[18] Zimlichman E, Henderson D, Tamir O, Franz C, Song P (2013). Health care-associated infections: a meta-analysis of costs and financial impact on the US health care system. JAMA Intern Med.

[19] Scott RD, Culler SD, Baggs J, Reddy SC, Slifka KJ (2024). Measuring the direct medical costs of hospital-onset infections using an analogy costing framework. PharmacoEconomics.

[20] Weber DJ, Rutala WA, Anderson DJ, Sickbert-Bennett EE (2023). Biofilms on medical instruments and surfaces: Do they interfere with instrument reprocessing and surface disinfection. Am J Infect Control.

[21] Azeredo J, Azevedo NF, Briandet R, Cerca N, Coenye T (2017). Critical review on biofilm methods. Biofouling.

[22] Sharma S, Mohler J, Mahajan SD, Schwartz SA, Bruggemann L (2023). Microbial biofilm: a review on formation, infection, antibiotic resistance, control measures, and innovative treatment. Microorganisms.

[23] Alfa MJ, Howie R (2009). Modeling microbial survival in buildup biofilm for complex medical devices. BMC Infect Dis.

[24] Alfa MJ, Ribeiro MM, da Costa Luciano C, Franca R, Olson N (2017). A novel polytetrafluoroethylene-channel model, which simulates low levels of culturable bacteria in buildup biofilm after repeated endoscope reprocessing. Gastrointest Endosc.

[25] Ribeiro MM, Graziano KU, Olson N, França R, Alfa MJ (2020). The polytetrafluoroethylene (PTFE) channel model of cyclic-buildup biofilm and traditional biofilm: the impact of friction, and detergent on cleaning and subsequent high-level disinfection. Infect Control Hosp Epidemiol.

[26] Wong M, Wang Y, Wang H, Marrone AK, Haugen SP (2020). Research: fluorescence microscopy-based protocol for detecting residual bacteria on medical devices. Biomed Instrum Technol.

[27] Anderson GG, James S, Kovvali S, Dang FW, Vishwakarma A (2026). Comparison of two models of biofilm formation on reusable stainless steel medical device material. J Hosp Infect.

[28] Goeres DM, Hamilton MA, Beck NA, Buckingham-Meyer K, Hilyard JD (2009). A method for growing a biofilm under low shear at the air-liquid interface using the drip flow biofilm reactor. Nat Protoc.

[30] Alfa MJ, Singh H (2020). Impact of wet storage and other factors on biofilm formation and contamination of patient-ready endoscopes: a narrative review. Gastrointest Endosc.

[31] Balan GG, Sfarti CV, Chiriac SA, Stanciu C, Trifan A (2019). Duodenoscope-associated infections: a review. Eur J Clin Microbiol Infect Dis.

[32] Kovaleva J (2017). Endoscope drying and its pitfalls. J Hosp Infect.

[33] Dong D, Thomas N, Ramezanpour M, Psaltis AJ, Huang S (2020). Inhibition of *Staphylococcus aureus* and *Pseudomonas aeruginosa* biofilms by quatsomes in low concentrations. Exp Biol Med.

[34] Pettit RK, Weber CA, Kean MJ, Hoffmann H, Pettit GR (2005). Microplate Alamar blue assay for *Staphylococcus* epidermidis biofilm susceptibility testing. Antimicrob Agents Chemother.

[35] Redelman CV, Maduakolam C, Anderson GG (2012). Alcohol treatment enhances *Staphylococcus aureus* biofilm development. FEMS Immunol Med Microbiol.

[36] Keogh D, Lam LN, Doyle LE, Matysik A, Pavagadhi S (2018). Extracellular electron transfer powers *Enterococcus faecalis* biofilm metabolism. mBio.

[37] Miao J, Lin S, Soteyome T, Peters BM, Li Y (2019). Biofilm formation of *Staphylococcus aureus* under food heat processing conditions: first report on CML production within biofilm. Sci Rep.

[38] Moormeier DE, Bose JL, Horswill AR, Bayles KW (2014). Temporal and stochastic control of *Staphylococcus aureus* biofilm development. mBio.

[39] Schaffer SD, Hutchison CA, Rouchon CN, Mdluli NV, Weinstein AJ (2023). Diverse *Enterococcus faecalis* strains show heterogeneity in biofilm properties. Res Microbiol.

[40] Frey AD, Kallio PT (2003). Bacterial hemoglobins and flavohemoglobins: versatile proteins and their impact on microbiology and biotechnology. FEMS Microbiol Rev.

[41] Webster DA, Dikshit KL, Pagilla KR, Stark BC (2021). The discovery of *Vitreoscilla* hemoglobin and early studies on its biochemical functions, the control of its expression, and its use in practical applications. Microorganisms.

[42] Benincasa M, Lagatolla C, Dolzani L, Milan A, Pacor S (2016). Biofilms from *Klebsiella pneumoniae*: matrix polysaccharide structure and interactions with antimicrobial peptides. Microorganisms.

[43] Cescutti P, De Benedetto G, Rizzo R (2016). Structural determination of the polysaccharide isolated from biofilms produced by a clinical strain of *Klebsiella pneumoniae*. Carbohydr Res.

[44] Ran SJ, Jiang W, Zhu CL, Liang JP (2015). Exploration of the mechanisms of biofilm formation by *Enterococcus faecalis* in glucose starvation environments. Aust Dent J.

[45] Sadovskaya I, Chaignon P, Kogan G, Chokr A, Vinogradov E (2006). Carbohydrate-containing components of biofilms produced in vitro by some staphylococcal strains related to orthopaedic prosthesis infections. FEMS Immunol Med Microbiol.

[46] Steinberger RE, Holden PA (2004). Macromolecular composition of unsaturated *Pseudomonas aeruginosa* biofilms with time and carbon source. Biofilms.

[47] Sugimoto S, Sato F, Miyakawa R, Chiba A, Onodera S (2018). Broad impact of extracellular DNA on biofilm formation by clinically isolated Methicillin-resistant and -sensitive strains of *Staphylococcus aureus*. Sci Rep.

[48] Rioufol C, Devys C, Meunier G, Perraud M, Goullet D (1999). Quantitative determination of endotoxins released by bacterial biofilms. J Hosp Infect.

[49] Wilson C, Lukowicz R, Merchant S, Valquier-Flynn H, Caballero J (2017). Quantitative and qualitative assessment methods for biofilm growth: a mini-review. Res Rev J Eng Technol.

[50] Alfa MJ, Degagne P, Olson N (1999). Worst-case soiling levels for patient-used flexible endoscopes before and after cleaning. Am J Infect Control.

[51] Alfa MJ (2019). Medical instrument reprocessing: current issues with cleaning and cleaning monitoring. Am J Infect Control.

[52] Chan ASF, Chan HLY, Yan BKL, Lai MKC (2023). Effectiveness of adenosine triphosphate to monitor manual cleaning and disinfection efficacy of flexible endoscopes in Hong Kong. JGH Open.

[53] Haas B, James S, Parker AE, Gagnon MC, Goulet N (2023). Comparison of quantification methods for an endoscope lumen biofilm model. *Biofilm*.

[54] Mandakhalikar KD, Rahmat JN, Chiong E, Neoh KG, Shen L (2018). Extraction and quantification of biofilm bacteria: method optimized for urinary catheters. Sci Rep.

[55] Herten M, Bisdas T, Knaack D, Becker K, Osada N (2017). Rapid in vitro quantification of *S. aureus* biofilms on vascular graft surfaces. Front Microbiol.

[56] Guan A, Wang Y, Phillips KS (2018). An extraction free modified o-phthalaldehyde assay for quantifying residual protein and microbial biofilms on surfaces. Biofouling.

