# Supplementary material for: Identifying markers of biofilm formation on medical-grade stainless steel as a representative medical device material
Source: Microbiology (Reading). 2026 Apr 1;172(4):001684. doi: 10.1099/mic.0.001684 (PMC13043183; doi:10.1099/mic.0.001684)
Supplement: Uncited Supplementary Material 1. [file mic-172-01684-s001.pdf]

SUPPLEMENTARY MATERIALS

Supplementary Table 1. Parameters for DFR experiments.

	Inoculum size (CFU/mL)	Batch phase time	Batch phase temperature	Continuous phase time	Continuous phase temperature
P. aeruginosa	3.65x10^7	6 hours	18-27°C	42 hours	18-27°C
K. pneumoniae	7.70x10^7				
S. aureus	4.15x10^7		35°C		
E. faecalis	1.73x10^7				

A

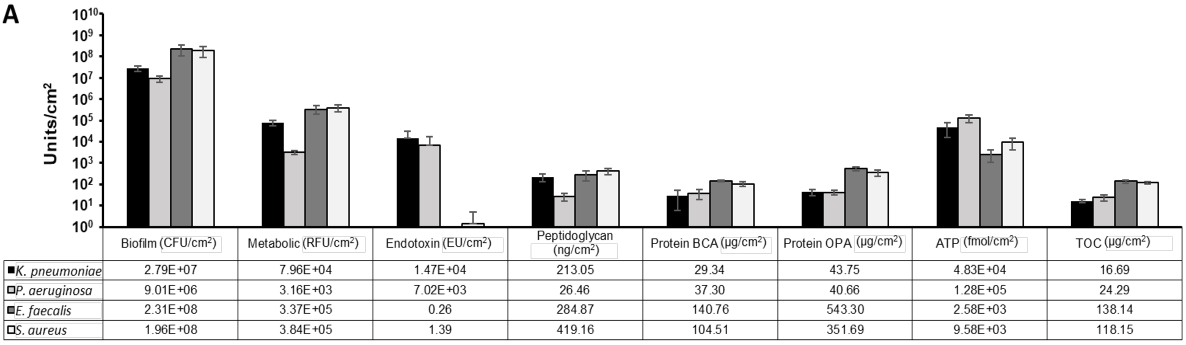

	Biofilm (CFU/cm²)	Metabolic (RFU/cm²)	Endotoxin (EU/cm²)	Peptidoglycan (ng/cm²)	Protein BCA (µg/cm²)	Protein OPA (µg/cm²)	ATP (fmol/cm²)	TOC (µg/cm²)
■ *K. pneumoniae*	2.79E+07	7.96E+04	1.47E+04	213.05	29.34	43.75	4.83E+04	16.69
□ *P. aeruginosa*	9.01E+06	3.16E+03	7.02E+03	26.46	37.30	40.66	1.28E+05	24.29
■ *E. faecalis*	2.31E+08	3.37E+05	0.26	284.87	140.76	543.30	2.58E+03	138.14
□ *S. aureus*	1.96E+08	3.84E+05	1.39	419.16	104.51	351.69	9.58E+03	118.15

B

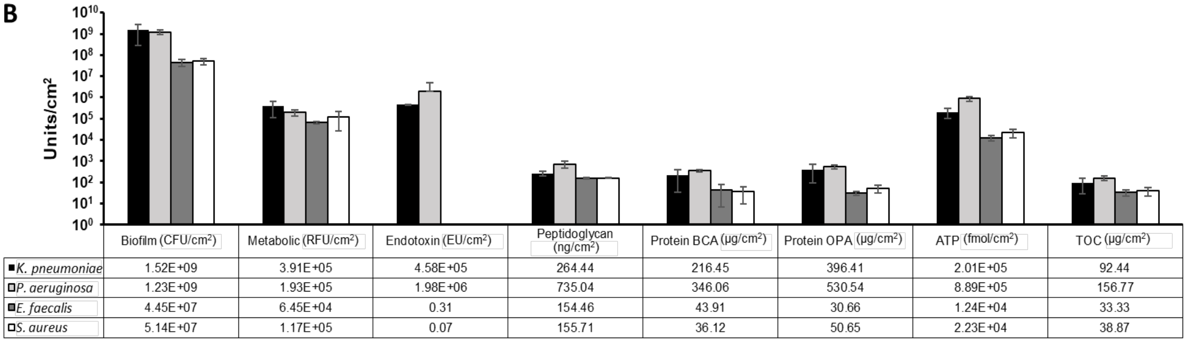

	Biofilm (CFU/cm²)	Metabolic (RFU/cm²)	Endotoxin (EU/cm²)	Peptidoglycan (ng/cm²)	Protein BCA (µg/cm²)	Protein OPA (µg/cm²)	ATP (fmol/cm²)	TOC (µg/cm²)
■ *K. pneumoniae*	1.52E+09	3.91E+05	4.58E+05	264.44	216.45	396.41	2.01E+05	92.44
□ *P. aeruginosa*	1.23E+09	1.93E+05	1.98E+06	735.04	346.06	530.54	8.89E+05	156.77
■ *E. faecalis*	4.45E+07	6.45E+04	0.31	154.46	43.91	30.66	1.24E+04	33.33
□ *S. aureus*	5.14E+07	1.17E+05	0.07	155.71	36.12	50.65	2.23E+04	38.87

Supplementary Figure 1. CFU and analyte levels of *P. aeruginosa, K. pneumoniae, S. aureus*, and *E. faecalis* biofilm extracts at 6 H (A) and 48 h (B). This figure consolidates CFU and analyte measurements presented in the main figures to facilitate comparison of relative biofilm burden and marker levels across species and growth stages. The average value of each marker is listed in the data tables. Error bars represent standard

deviation from three independent biological replicates.

Supplementary Table 2. Comparison of ST98 criteria, LOD, and marker levels.

Analyte	ST98 criteria	LOD (unit/cm^2) Per Test Conditions	Lowest Marker Value Obtained
**ATP**	≤ 22 fmol/cm^2	0.005 fmol/cm^2	2.6x10^3 fmol/cm^2
**Protein: OPA**	≤ 6.4 μg/cm^2	6.9 μg/cm^2	31 μg/cm^2
**Protein: BCA**	≤ 6.4 μg/cm^2	52.3 μg/cm^2	29 μg/cm^2
**TOC**	≤ 12 μg/cm^2	1.3 μg/cm^2	17 μg/cm^2
**Peptidoglycan**	N/A	0.005 ng/cm^2	27 ng/cm^2
**Endotoxin***	N/A	0.013 EU/cm^2	7x10^3 EU/cm^2

*****Endotoxin is only assessed for Gram-negative bacteria
